# Molecular recognition of ternary complexes: a new dimension in the structure-guided design of chemical degraders

**DOI:** 10.1042/EBC20170041

**Published:** 2017-11-08

**Authors:** Scott J. Hughes, Alessio Ciulli

**Affiliations:** Division of Biological Chemistry and Drug Discovery, School of Life Sciences, University of Dundee, Dow Street, Dundee DD1 5EH, Scotland, U.K.

**Keywords:** molecular glues, protein degradation, protein-protein interactions, PROTACs, ternary complexes

## Abstract

Molecular glues and bivalent inducers of protein degradation (also known as PROTACs) represent a fascinating new modality in pharmacotherapeutics: the potential to knockdown previously thought ‘undruggable’ targets at sub-stoichiometric concentrations in ways not possible using conventional inhibitors. Mounting evidence suggests these chemical agents, in concert with their target proteins, can be modelled as three-body binding equilibria that can exhibit significant cooperativity as a result of specific ligand-induced molecular recognition. Despite this, many existing drug design and optimization regimens still fixate on binary target engagement, in part due to limited structural data on ternary complexes. Recent crystal structures of protein complexes mediated by degrader molecules, including the first PROTAC ternary complex, underscore the importance of protein–protein interactions and intramolecular contacts to the mode of action of this class of compounds. These discoveries have opened the door to a new paradigm for structure-guided drug design: borrowing surface area and molecular recognition from nature to elicit cellular signalling.

## It takes two to tango: the classical binary approach to drug discovery

Protein structure is firmly regarded as a fundamental cornerstone of drug discovery platforms. Over the last quarter century, this importance has been magnified by advancements in biophysical techniques, including X-ray crystallography, nuclear magnetic resonance (NMR) spectroscopy, surface plasmon resonance (SPR) and isothermal titration calorimetry (ITC), and more recently the advent of cryo-electron microscopy (cryo-EM). Despite the contribution these advances have made to furthering drug discovery initiatives, structure-based drug design (SBDD) is still largely exemplified by approaches developed following the first structures of haemoglobin [1,2], renin [3,4] and the HIV protease [5,6]. This classical approach was often active-site directed, and involved large-scale screening of chemical libraries to identify compounds that elicit a desired activity against a biological target. Although the advent of modern genomics and combinatorial chemistry fuelled an increase in the number of known biological targets and available compounds, this did not translate to as many new drugs reaching the marketplace as was initially hoped for [7]. Even with restrictions imposed by Lipinski’s ‘rule of five’ [8], the number of possible chemical compounds to screen from is estimated at approximately >10^63^ [9].

A desire to reduce the breadth of chemical space available for drug screening motivated and underpinned the emergence of fragment-based drug discovery (FBDD), which typically relies on screening and evaluating compounds with mass <250 Da [7]. Although early efforts in FBDD relied heavily on ligand-based or protein-based NMR to detect binding, X-ray crystal structures are necessary to guide appropriate growth and/or linking of fragment hits into optimized molecules. Most modern FBDD platforms employ a mixture of NMR, X-ray crystallography and various biophysical/biochemical techniques for monitoring the typically weak interactions seen with fragments [10].

Whereas FBDD sought to reduce the size of chemical libraries for screening, many drug platforms, until recently, suffered from a low number of druggable targets. Here druggability refers to targets with a well-defined binding site – notably members of conventional target classes such as protein kinases and GPCRs – which can be targeted with intermediate or transition-state analogues as well as low-molecular weight drug-like small molecules [6]. To expand the scope of the druggable genome, numerous drug discovery programmes now aim to exploit protein regulatory elements, including allosteric sites and surfaces that mediate interactions between two or more proteins.

## Molecular glues and the modulation of protein–protein interactions

**Figure 1:**
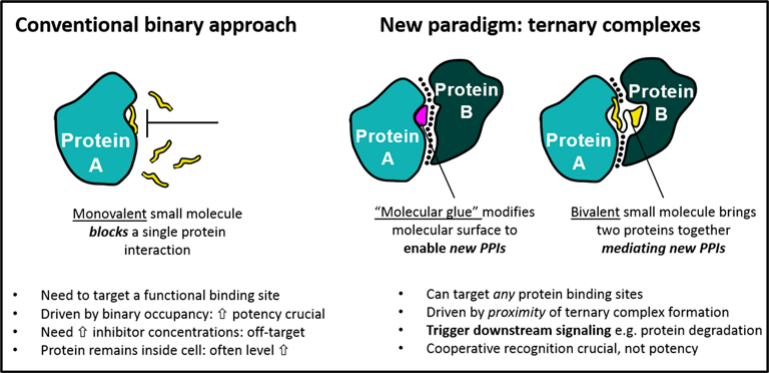
Comparison of binary and ternary therapeutic approaches

An increasing understanding of the role of protein–protein interactions (PPIs) has paved the way for a new paradigm in molecular therapeutics, one that focuses on agents that stabilize/induce interactions between proteins, rather than disrupting them or blocking their enzymatic activity [11,12]. In contrast with conventional active-site directed inhibition, these so-called PPI modulators bind protein surfaces or interfaces and stabilize interaction(s) with a second protein, ultimately resulting in the activation or suppression of a cellular response [13]. Although targeting PPIs can prove difficult due to the large, shallow, and often solvent-exposed surfaces that comprise them, PPI modulators are attractive to drug development. Firstly, compounds stabilizing PPIs often do not need to compete with endogenous ligands, allowing some leeway in the potency required for biological activity. Secondly, and perhaps more importantly, PPI modulators have the potential to target proteins deemed undruggable because it is not necessary to target a conventional active site. Several key differences between the conventional binary approach and the new ternary approach provided by PPI modulators – here sub-classified as molecular glues and bivalent ligands – are shown in Figure 1.

Molecular glues refer to small-molecule PPI stabilizers that bind a protein and modify its molecular surface, thus enabling it to recruit a new protein (Figure 1) [14]. Several examples of molecular glues can be found in nature, including some macrocyclic agents initially thought to act via conventional inhibition. The immunosuppressive drug cyclosporin A (CsA) was originally identified as an inhibitor of the peptidyl–prolyl isomerase cyclophilin A (CyPA), but it was later discovered that the immunosuppressive activity results from interactions between ligand-isomerase CyPA:CsA complex and calcineurin A (CaN), a Ser/Thr phosphatase involved in T-cell activation. In this *de novo* complex, the CsA forms interactions with both CyPA and a homodimer of CaN, the net result of which is to block the catalytic site of CaN [15–18]. Similarly, the macrolide rapamycin has been shown to promote binding of the mammalian target of rapamycin (mTOR) FRB domain and the immunophilin FKBP12. The resulting ternary complex of FRB:rapamycin:FKBP12 shows *de novo* interactions between the FRB domain and FKPB12 in addition to interactions between rapamycin and both proteins [19,20]. The precise mechanism of action is still unknown, but it is suggested that FKB12 may impede access to the mTOR catalytic site [14].

## Molecular glues hijacking E3 ubiquitin ligases

A rapidly growing application of PPI modulation is targeting proteins for degradation by the proteasome. This approach typically involves hijacking the activity of specific Cullin RING E3 ubiquitin ligases (CRLs), the largest family of E3 ligases in the ubiquitin–proteasome system (UPS) that serve as scaffolds between a substrate protein and a ubiquitin-conjugating enzyme (E2) [21]. This allows the transfer of ubiquitin (Ub) to the substrate, thereby initiating the proteasomal degradation of the latter. Modulation of E3 activity is observed with several natural molecules of varying size and character, including the plant hormones auxin and jasmonate, and viral proteins such as Vif and SV5. In both cases, the catalytic proximity-driven activity of CRLs is hijacked by the molecular glue in order to target a protein for degradation [14,22–24]. Plants use auxin to promote the ubiquitin-dependent degradation of Aux/IAAs, a family of proteins that regulate the activity of the auxin responsive factor (ARF) family of transcription factors. The precise mechanism behind this targeted degradation involves an auxin-dependent interaction between the SCF–TIR1 ubiquitin ligase complex and the Aux/IAA recognition motif. Here, auxin binds to TIR1 and provides the contact surface for which Aux/IAA can bind [25,26].

**Figure 2:**
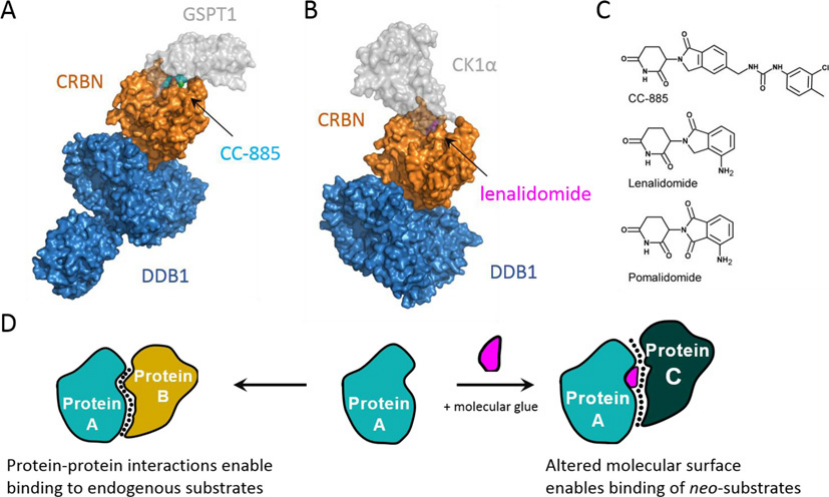
Molecular glues (**A**) Crystal structure of DDB1:CRBN:CC-885:GSPT1 (PDB ID: 5HXB) [36] and (**B**) crystal structure of DDB1:CRBN:lenalidomide:CK1α (PDB ID: 5FQD) [37]. (**C**) Chemical structures of several IMiDs. (**D**) Schematic illustrating the modification of binding surfaces by molecular glues.

The immunomodulatory drugs (IMiDs), including the infamous teratogenic thalidomide and its second-generation derivatives pomalidomide and lenalidomide, have also been identified as acting as molecular glues (Figure 2C). Despite their sullied history, these early IMiDs have been approved for the treatment of multiple myeloma due to their anti-proliferative, anti-angiogenic and immunomodulatory properties [27–29]. Indeed, lenalidomide (trade name Revlimid) is currently the top oncology product 2017 according to IgeaHub (https://igeahub.com/2017/09/23/top-30-oncology-drugs-2017/), and is expected to be the top-selling cancer drug by 2022 (FiercePharma: http://www.fiercepharma.com/special-report/special-report-top-15-best-selling-cancer-drugs-2022). However, the mode of action of the IMiDs remained elusive until 2010, when the molecular target was identified as cereblon (CRBN), the substrate receptor that forms part of the CUL4–Rbx1–DDB1–CRBN E3 ubiquitin ligase complex (CRL4^CRBN^) [29,30]. The resulting CRBN–IMiD complex is able to form novel protein–protein interactions with several proteins, including the Ikaros (IKZF1) and AIOLOS (IKZF3) transcription factors [31,32] and CK1α [33]. As a result of this small-molecule mediated recognition event, these proteins become so-called *neo*-substrates to the CRBN E3 ligase, resulting in their ubiquitination and subsequent degradation. Interestingly, only lenalidomide leads to the degradation of CK1α, suggesting that variations in phthalimide group present in the different IMiDs results in distinct modifications to the molecular surface of CRBN and thus differing preferences for substrate recognition (Figure 2D).

Crystal structures of DDB1–CRBN bound to thalidomide, pomalidomide and lenalidomide were solved and published in 2014 [34,35]. These structures help to explain this difference in CRBN E3 ligase substrate specificity. The glutarimide moieties of all the IMiDs bind to a hydrophobic pocket defined by three tryptophan residues – called the ‘thalidomide-binding pocket’ – within CRBN. In contrast, the phthalidomide/isoindolone rings are solvent exposed and alter the molecular surface of CRBN, thereby modulating substrate recognition. Two recent ternary structures of DDB1–CRBN in complex with IMiD-like molecules and *neo*-substrate protein provide a unique look at *de novo* binding interfaces that result from different molecular glues with different substrates [36,37]. A structure of CRBN–lenalidomide with bound CK1α (PDB code: 5FQD; Figure 2B) confirms that the small molecule is required for CK1α binding, as both CRBN and lenalidomide form interactions with a key β-hairpin loop of CK1α [37]. A second structure describes the ternary complex between CRBN, lenalidomide analogue compound CC-885 and *neo*-substrate protein GSPT1 (PDB code: 5HXB; Figure 2A) [36]. CC-885 is a newer generation IMiD and the first to display anti-tumour activity in both epithelial and haematological cancers. Although CC-885 is able to induce CRBN-mediated degradation of Ikaros, its anti-tumour properties stem from *de novo* interactions between CRBN–CC-885 and the translation termination factor GSPT1. Indeed, the crystal structure of the ternary complex reveals that a surface turn on GSPT1 interacts with both CC-885 and a ‘hotspot’ on CRBN [36]. Comparison of the two ternary crystal structures reveal the striking similarities in shape between the β-hairpin of the two *neo*-substrates, despite poor sequence identity. Structural comparison also identified conservation of a key Gly residue at the interface with the molecular glue, which was confirmed by site-directed mutagenesis. The interactions between GSPT1 and the extended urea moiety of CC-885 may also explain the selectivity, as both lenalidomide and pomalidomide lack this moiety. Interestingly, the two *neo*-substrates dock to the ligase from distinct directions, highlighting the permissivity of E3 ligases to diverse spatial orientations and presentations of *neo*-substrates for ubiquitination [38].

## Proteolysis-targeting chimaeras (PROTACs): a bivalent approach to PPI modulation

**Figure 3:**
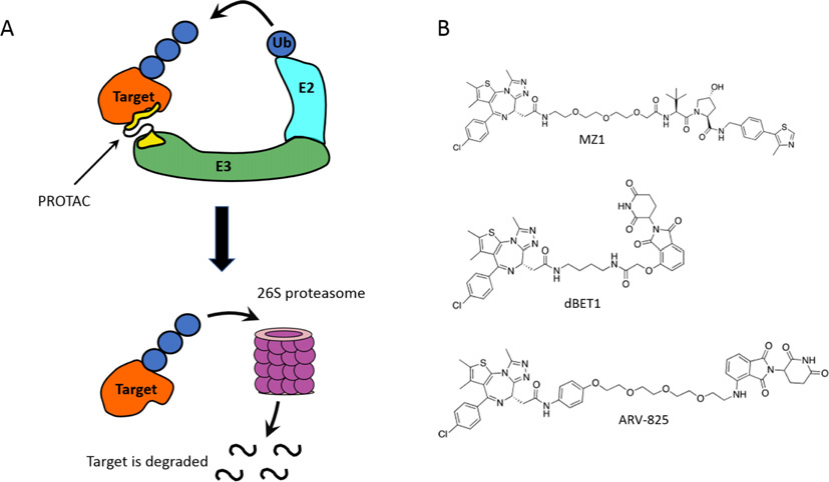
Proteolysis-targeting chimaeras (**A**) PROTACs hijack E3 ligases to trigger the ubiquitination and subsequent proteasomal degradation of a target protein. Ub - ubiquitin. (**B**) Chemical structures of PROTACs that recruit BET proteins and either VHL (MZ1) or CRBN (dBET1 and ARV-825).

Although the molecular glues can modify the substrate affinity and selectivity profiles of an E3 ligase, typically they are found to bind either weakly to one or both of the two proteins individually or to one protein alone, usually the E3 ligase. By contrast, a bivalent approach to protein degradation contemplates two-headed small molecules that are able to bind to each of the two proteins individually. A synthetic and potentially modular bivalent approach to promoting selective degradation of a target protein was proposed in 2001 by Crews, Deshaies and Sakamoto, which was named ‘Proteolysis-Targeting Chimaeras’ (PROTACs) [39]. PROTACs are heterobifunctional compounds that are designed to bind both a target protein and an E3 ligase at the same time, via two separate heads, to target a particular protein for degradation by the proteasome (Figure 3A). Due to the scarcity of suitable ligands for E3 ligases, the first-generation PROTACs utilized known peptidic epitopes of E3 ligase substrates. For example, a portion of NF-κB inhibitor-α containing phosphor-serine as key recognition degron was used to recruit the S-phase kinase-associated protein 1 (SKP1)–cullin 1-F-box E3 ligase complex (SCF^bTRCP^) [40]. This was later replaced with the minimal recognition sequence ALA–Hyp–YIP, from the hypoxia-inducible factor 1α (HIF-1α), which specifically binds to the von Hippel–Lindau (VHL) Cullin2 E3 ligase (CRL2^VHL^) [41]. The major limitations of these early peptide-based PROTACs were their peptidic nature and high molecular weight, meaning the compounds suffered from poor cell permeability, low cellular stability and, consequently, limited cellular activity [42]. To address these limitations, it was recognized the need to replace such peptidic E3-targeting ligands with small molecules of lower molecular weight and more drug-like properties.

A considerable leap forward in the field came out of the development of non-peptidic drug-like small-molecule binders of VHL with crystallographically defined binding modes. A first-generation of VHL ligands with micromolar binding affinities to VHL were reported in 2012 as the product of a collaboration between the Ciulli (Cambridge) and the Crews (Yale) laboratories [43–45]. Extensive and careful SBDD and FBDD optimization around the key hydroxyproline residue, supported by co-crystal structures and biophysics-driven SAR, ultimately led to the discovery of potent ligands with binding affinity in the nanomolar range. This series was later optimized within the Ciulli lab (Dundee) into compound VH298, a highly potent, selective and cell-active VHL inhibitor in its own right [46–48]. These potent and drug-like VHL ligands allowed the PROTAC field to begin to move from an initial chemical biology proof-of-concept, into the realm of lead discovery for therapeutic purposes. Amongst the first PROTACs to make use of these non-peptidic VHL ligands – MZ1 (first VHL-based BET degrader disclosed in 2015 by the Ciulli Lab [49], see Figure 3B) and ARV-771 (2016, from Yale spin-off Arvinas [50]) – were conjugates of JQ1, a pan-selective inhibitor of bromo- and extra-terminal (BET) domain family of proteins (*K*_d_ ∼ 100 nM equally against the bromodomains of BET proteins Brd2, Brd3 and Brd4). Degradation of BET proteins was observed in several cell lines following treatment with VHL-based BET degraders. Notably, MZ1-mediated degradation of BET proteins in cancer cell lines was shown to be preferential for Brd4 over paralogous proteins Brd2 and Brd3 [49], with an observed selectivity window of >10-fold in protein degradation activity, or DC50, for Brd4 [51]. This observation was intriguing and important, as it demonstrated for the first time an added layer of target depletion selectivity over the binary target engagement selectivity of the target warhead ligand. As the derivatization of JQ1 into MZ1 did not result in differences in target engagement selectivity with the different BET bromodomains, it was postulated that the choice of the E3 ligase recruited could play an important role, together with the conjugation position and choice of linker, in dictating the enhanced target degradation selectivity. Indeed, differential target depletion selectivities were later reported with PROTAC molecules designed to target the Abl kinase proteins to degradation via conjugation of CRBN and VHL targeting ligands [52]. Moreover, BET-targeting PROTACs recruiting CRBN were reported at the same time of MZ1 and were shown to be pan-selective degraders of all BET proteins at nanomolar concentrations [53,54]. Two compounds, ARV-825 (from Arvinas) and dBET1 (from the Bradner Lab at Dana Farber), were designed as conjugates of a pomalidomide derivative and an analogue of JQ1 (Figure 3B), and induced complete degradation of Brd2, Brd3 and Brd4 in different cell lines. Not only could highly potent PROTACs now be designed to be active in cells at concentrations that were orders of magnitudes lower than the binary binding affinities of the constitutive warheads, but they could also exhibit exquisite target degradation selectivities.

## The bicameral design and optimization of PROTACs

Despite the ever-growing number of targets recruited to E3 ligases using PROTACs, the majority of them are derived using classical binary SBDD approaches – similar to the pioneering examples targeting BET bromodomains and protein kinases, described above. In many cases, existing inhibitors/binders that may or may not have therapeutic efficacy on their own are being re-purposed as PROTACs. This has led to the design of VHL-recruiting PROTACs that target c-ABL, RIPK2, BET proteins and ERRα [49,50,52,55], and CRBN-recruiting PROTACs that target BET proteins, FKBP12 and c-ABL [52–54]. Other E3 ligases that have been recruited by PROTACs include cIAP, also termed specific and non-genetic IAP-dependent protein erasers (SNIPERs) [56,57], and MDM2 [58].

**Figure 4:**
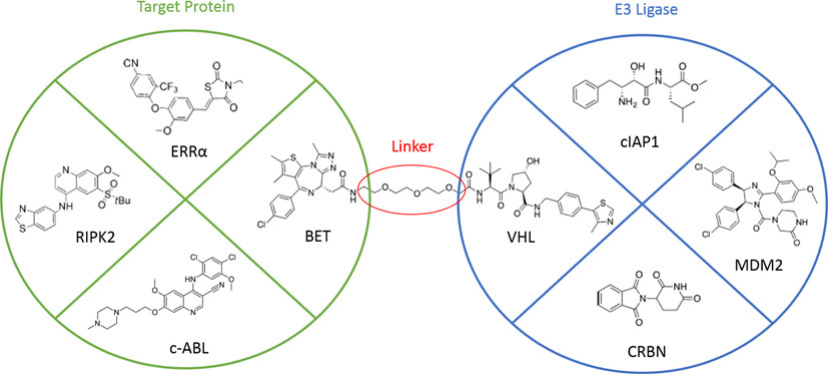
Existing mindset in PROTAC design and optimization The prevailing approach to developing new PROTACs views ligands targeting the E3 Ligase (blue) and the Target Protein (green) as separate entities that can be joined by a linker.

To date, the design and subsequent optimization of PROTACs have been largely guided by a fixation on the binary interactions between each protein and the respective warhead of the PROTAC (Figure 4). This is driven by the abundance of crystal structures of binary complexes, many of which are available in the Protein Data Bank (PDB). However, recent studies have demonstrated the drawbacks to this approach, as improved binary affinity does not necessarily translate to improved degradation of target protein [51]. Initially, linkers were little more than a means to conjugate two ligands together to form a PROTAC. Typical linker design employs varying lengths of polyethylene glycol (PEG), but there are some notable examples that deviate from this template, including the pure alkyl chains of dBET1 and dFKBP-1 [54]. The site of attachment is often based on analysis of ligand-bound structures, where available, to look for regions that are solvent exposed. In this way, linker attachment was handled much like biotinylation or the addition of a fluorophore. However, several studies have attempted to systematically examine linker properties, including length and attachment site, and have demonstrated that they do influence protein degradation. ER-degrading PROTACs that recruit VHL were shown to promote greater degradation with an optimal linker length of 16 atoms [59]. Degradation was also enhanced when the linker was derivatized from the C-7a position of oestradiol rather than via ester linkage to O-17 [42]. In this latter case, the enhanced degradation may stem from the carbon–carbon derivatives’ reduced susceptibility to esterases. Other studies have explored selective coupling chemistries e.g. click chemistry to assemble PROTAC in cell and as libraries [60,61]. More recently, the role of linker length in PROTACs that degrade TANK-binding kinase 1 (TBK1) was investigated [62]. Once again, the study demonstrated remarkable differences in degradation solely due to varied linker length. Thus, although linker properties clearly have a marked effect on the ability for PROTACs to induce degradation, how this occurs has remained poorly understood.

Taken together, mainstream approaches to PROTAC design have so far appeared to view complex formation as two binary interactions. Warheads are initially designed and optimized against their respective target, then conjugated using linkers that vary in length and flexibility. Multiple derivation sites from each ligand may also be explored to further expand the PROTAC series. While these three stages have yielded potent PROTACs that promote degradation of a variety of protein targets, the process still had remained somewhat unguided at the level of structural understanding of the key catalytic ternary complex. Moreover, no rational approach to the design of linkers seems to include the possibility of inter- and intramolecular interactions. However, similar to the CRBN–IMiD ternary structures described above, the linker may be able in many cases to interact favourably with both proteins, the warheads and, given an appropriate length and flexibility, even with itself.

We argue that the binary plug-and-play approach to PROTAC design (Figure 4) has been largely the result of a major limitation in the field – the lack of structural data regarding the ternary complex. Although there are some examples of crystallizing PROTACs bound to one of their target proteins in a binary complex e.g. Brd4/DB-2-190 (PDB ID: 4ZC9) [54], these structures are not guaranteed to capture the correct conformation of the linker/second warhead due to the absence of the partner protein. *In silico* modelling of the ternary structure has been attempted, as in the case of the CRBN:PROTAC:SIRT2 complex [63] amongst others, but again this may not mirror reality due to the difficulty in accurately predicting protein–protein complexes computationally, let alone complexes that are formed only in the presence of an artificial ligand.

The molecular glue approach exemplified by the IMiD-mediated recruitment of CRBN demonstrates the requirement to promote *de novo* PPIs between the two targeted proteins in the ternary complex. In contrast, the bivalent nature of PROTACs with two independent ligands present in the same molecular entity was deemed to imply that PROTACs would merely act by bringing the two proteins into spatial proximity, in a fashion independent of protein–protein interactions. This incorrect notion was soon to be debunked as the first structural image of a PROTAC-mediated ternary complex emerged in early 2017.

## The whole is greater than the sum of the parts: emergence of a ternary model

**Figure 5:**
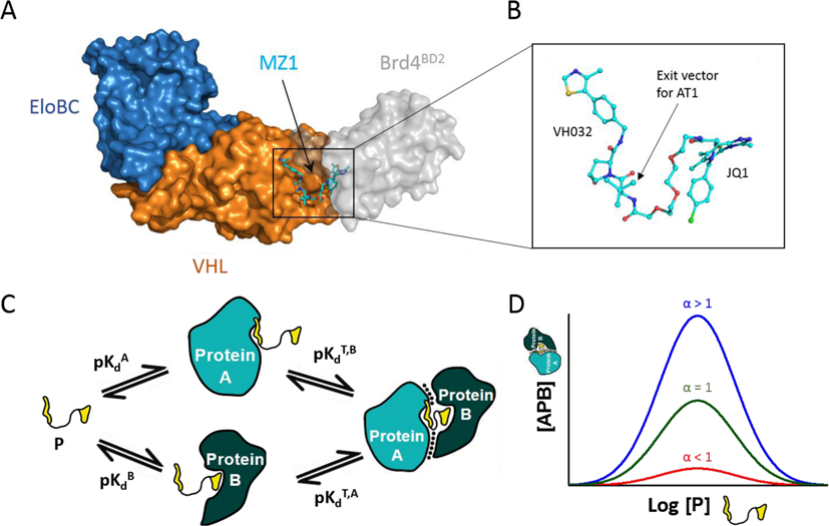
PROTAC-induced ternary complexes and their biophysical properties (**A**) Crystal structure of VHL:MZ1:Brd4^BD2^ (PDB ID: 5T35). (**B**) Conformation of MZ1 when bound to the ternary complex. (**C**) Ternary (T) complex equilibria for protein A, protein B and PROTAC (P). (**D**) Simulation showing amount of ternary complex formation and its relationship with cooperativity. *K*_d_^A^ and *K*_d_^B^ are maintained constant for all three cases.

In a recent crystal structure of PROTAC MZ1 bound in complex with both VHL and the Brd4 bromodomain (Brd4^BD2^), Gadd et al. [64] provide the first glimpse into a PROTAC-induced ternary species, and a complete PROTAC binding site (Figure 5A). MZ1 binds to a bowl-shaped pocket formed by extensive PPIs between VHL and Brd4^BD2^. The base of the bowl arises from two sets of non-polar interactions, whereas the rim of the bowl is largely formed through complementary electrostatic interactions and salt bridges between side chains on Brd4^BD2^ and VHL. The total buried surface area for the ternary complex, including PPIs and surface area buried due to ligand folding, was estimated at >2600 Å^2^. For comparison, the total buried surface areas in CRBN:lenalidomide:CK1α and CRBN:CC-885:GSPT1 structures were estimated at 1830 Å^2^ and 2390 Å^2^, respectively.

In addition to the JQ1 and VH032 warheads forming expected binary interactions with their respective protein target, MZ1 also forms a variety of inter- and intramolecular contacts within the ternary complex. The PEG linker is heavily buried and forms interactions with both Brd4^BD2^ and VHL, resulting in very little solvent-exposed linker compared with many prevailing models at the time. Similarly, regions from each warhead that are solvent-exposed in the respective binary complexes are instead buried in the ternary complex. This may play a key role in stabilizing the complex, as JQ1 forms interactions with VHL and VH032 forms interactions with Brd4^BD2^. Beyond the extensive protein–ligand interactions, the linker also folds up to allow sandwiching between JQ1 and VH032. Due to this linker collapse, it was posited that the points of linkage could be optimized so as to rigidify the PROTAC and reduce its conformational freedom. Given the proximity between the *tert*-Leu group of VH032 and the JQ1 ligand, this position was chosen for a new linkage (Figure 5B). The resulting PROTAC series, AT1-6, displayed enhanced selectivity for Brd4, albeit with reduced potency relative to MZ1 due to a slight reduction of binding affinity at the VHL warhead [64].

The ternary complex structure also provided, crucially, insights towards an explanation for the preference for Brd4 degradation by MZ1 despite the pan-selective properties of JQ1. Specifically, the ternary structure suggested that selectivity could be governed by cooperativity, itself a by-product of isoform-specific interactions. For ternary complex [APB], cooperativity (represented as *α*) is a metric to describe favourable or repulsive interactions between ‘A’ and ‘B’ in the ternary complex. To measure cooperativity, Gadd et al. developed reversed ITC experiments to measure *K*_d_^A^ and *K*_d_^T,A^ (Figure 5C) and thus determine to what extent the binding of protein A to a PROTAC ‘P’ is altered by the presence of protein B [64]. Negative cooperativity, here defined as *α* = *K*_d_^A^ / *K*_d_^T,A^ < 1, occurs when interactions between A and B diminish the amount of ternary complex APB. Conversely, positive cooperativity, defined as *α* = *K*_d_^A^ / *K*_d_^T,A^ > 1, occurs when interactions between A and B enhance complex formation. All the BET bromodomains showed positive cooperativity, with the strongest cooperativity observed for the crystallized target: Brd4^BD2^. Furthermore, strength of positive cooperativity and the amount of ternary complex formed experimentally were found to be highly correlated (Figure 5D). Reciprocal mutations in residues at the VHL–bromodomain interfaces of Brd4^BD2^ and Brd2^BD1^– the bromodomains displaying the highest and lowest cooperativities, respectively – completely reversed the biophysical phenotypes for each bromodomain, further suggesting a tight correlation between PPIs, cooperativity and ternary complex formation. Importantly, the mutations did not affect the binary affinity for MZ1, demonstrating that the change in cooperativity was not due to a change in binary target engagement [56].

## Three’s company: the way forward

**Figure 6:**
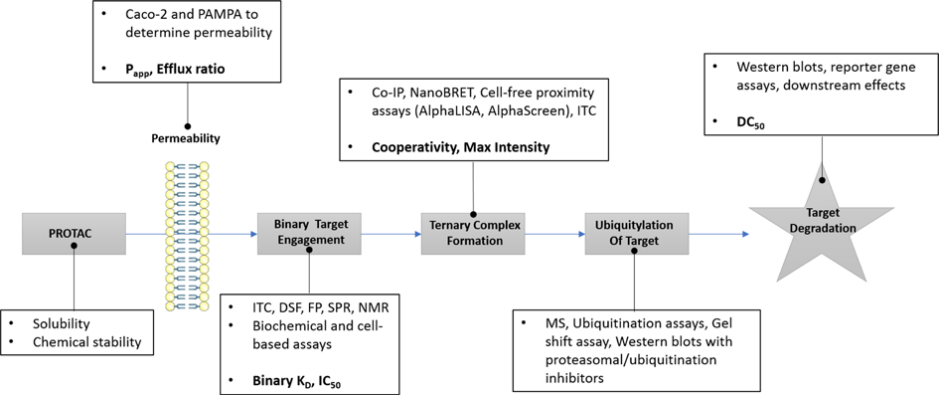
The process of PROTAC development The chart highlights the various steps involved from administration to intracellular degradation and the biophysical techniques used to study them. In bold are common parameters to characterize PROTACs at each stage.

The future of PROTAC development must undoubtedly include ternary system thermodynamic and kinetic properties, including cooperativity, as well as an appreciation for the ternary structure as a whole. The PROTAC developmental process can be illustrated as the path a PROTAC must follow from administration to intracellular degradation, with each stage characterized by specific parameters that can be monitored using tailored assays and techniques (Figure 6). Briefly, the process begins with important regard for the chemical stability, solubility and permeability of the PROTAC. Once intracellular, the PROTAC will undergo binary engagement with either the E3 ligase or the target protein; this interaction can be described using IC_50_ values and *K*_d_ values, amongst other binary kinetic and thermodynamic parameters, which can be obtained through biophysical techniques such as ITC, fluorescence polarization (FP), SPR and BLI. While it is clear that binary target engagement primes ternary complex formation, characterizing the ternary system is an additional important step. X-ray crystal structures of the ternary complex at this stage will prove invaluable, but many other tools that have a prominent role in existing drug discovery platforms can, or have the potential to, also be adapted to assess the properties of ternary systems, as depicted in Figure 6 and detailed in Table 1. For example, ITC, as discussed above, is in our experience the most reliable technique for determining cooperativity, but we envisage that other binding assays could be developed or configured for the same purposes. Table 1 highlights how various gold-standard biophysical techniques can be repurposed to interrogate ternary systems. A recent study from our lab has reported the application of ITC, size exclusion chromatography and AlphaLISA in combination to monitor the 2:1 complexes formed between homo-bivalent VHL degrading molecules (also called Homo-PROTACs) and the target protein VHL [65]. Crucially, the most potent VHL degrader, compound CM11, was found to induce VHL dimerization with the greatest cooperativity effect, further underscoring the importance of this parameter in driving ternary complex equilibria and PROTAC degradation efficiency [65 ]. Following ternary complex formation, it is expected that the target will be ubiquitinated and targeted to the proteasome for degradation. These processes can be monitored through a variety of biochemical approaches, including mass spectrometry and Western blot assays following treatment of cells with PROTACs in the presence and absence of inhibitors of ubiquitination and the proteasome. Establishing a link between ternary complex formation and degradation is also warranted, as it illustrates the importance of PROTAC geometry and the orientation of the proteins relative to one another.

**Table 1:** Application of biophysical techniques to study binary and ternary complex formation

Technique	Binary	Ternary
X-ray	• Identify ligand binding site and binding mode	• Identify folding and conformation of the bound PROTAC, particularly the linker
	• Compound optimization through visualization of protein–ligand interactions	• Identify *de novo* protein–protein interactions to understand *neo*-substrate binding mode relative to E3 ligase
		• Visualization of inter- and intramolecular interactions made by PROTAC enables further optimization
NMR	• Ligand-observed NMR can provide information on bound ligand conformation	• Ligand-observed NMR could provide information on bound conformation of PROTAC in ternary complex
	• Protein-observed can map binding site on protein	• Protein-observed can map protein–protein contacts in the ternary complex
SPR/BLI	• Binary *K*_d_ values. *k*_on_ and *k*_off_ rate constants	• Ternary *K*_d_ values. *k*_on_ and *k*_off_ rate constants for the dissociation of the ternary complex
DSF	• Small-molecule hit identification	• Applicability remains to be demonstrated
ITC	• Binary *K*_d_ values, thermodynamic parameters for a binary system i.e. Δ*H*, Δ*S* and Δ*C*_p_ of binding	• Ternary *K*_d_ values and cooperativity; thermodynamic parameters for a ternary system; ternary complex stabilities (Δ*G*)
AlphaLISA	• Competition assays for hit validation	• Assess capacity for forming ternary complex; evaluate relative population of ternary complexes
		• Generate bell-shaped curves typical for three-body binding equilibria
FP	• Competition assays for hit validation	• Applicability to be demonstrated
	• Binary *K*_d_ values can be back-calculated from IC_50_ values	
TR-FRET	• Competition assays for hit validation	• Assess capacity for forming ternary complex; evaluate relative population of ternary complexes
		• Generate bell-shaped curves typical for three-body binding equilibria

## Summary

- PROTACs and molecular glues are a rapidly growing application of PPI modulation that target proteins for degradation by the proteasome.
- PROTACs and molecular glues represent a new paradigm in therapeutics, capable of targeting any binding site and driven by ternary complex formation.
- Recent elucidation of the ternary complex structures reveals extensive inter- and intramolecular interactions formed by the bound ligand that cannot be predicted solely from binary target engagement.
- The future of PROTAC development must include ternary system thermodynamic and kinetic properties, including cooperativity, as well as an appreciation for the ternary structure as a whole.

## Abbreviations

BET: bromo- and extra-terminal
CaN: calcineurin A
CK1α: Casein Kinase 1α
CRBN: cereblon
CRL: Cullin RING E3 ubiquitin ligase
CsA: cyclosporin A
CyPA: cyclophilin A
FBDD: fragment-based drug discovery
GSPT1: G1 To S Phase Transition 1
HIF-1α: hypoxia-inducible factor 1α
IMiD: immunomodulatory drug
ITC: isothermal titration calorimetry
MDM2: Mouse double minute 2 homolog
NF-κB: Nuclear Factor - κB
PEG: polyethylene glycol
PPI: protein–protein interaction
SBDD: structure-based drug design
SCF: SKp, Cullin, F-box containing complex
SKP1: S-phase kinase-associated protein 1
SPR: surface plasmon resonance
TIR1: Transport Inhibitor Response 1
UPS: ubiquitin–proteasome system
VHL: von Hippel–Lindau
